# Childhood Illness Prevalence and Health Seeking Behavior Patterns in Rural Tanzania

**DOI:** 10.1186/s12889-015-2264-6

**Published:** 2015-09-23

**Authors:** Almamy M. Kanté, Hialy R. Gutierrez, Anna M. Larsen, Elizabeth F. Jackson, Stéphane Helleringer, Amon Exavery, Kassimu Tani, James F. Phillips

**Affiliations:** Mailman School of Public Health, Columbia University, 60 Haven Avenue, New York, 10032 USA; Ifakara Health Institute, PO Box 78373, Dar es Salaam, Mikocheni Tanzania; Swiss Tropical and Public Health Institute, Socinstrasse 57, Basel, CH-4002 Switzerland; University of Basel, Petersplatz 1, Basel, CH-4003 Switzerland; Bloomberg School of Public Health, Johns Hopkins University, 615 N. Wolfe Street, Baltimore, MD 21205 USA

## Abstract

**Introduction:**

This paper identifies factors influencing differences in the prevalence of diarrhea, fever and acute respiratory infection (ARI), and health seeking behavior among caregivers of children under age five in rural Tanzania.

**Methods:**

Using cross-sectional survey data collected in Kilombero, Ulanga, and Rufiji districts, the analysis included 1,643 caregivers who lived with 2,077 children under five years old. Logistic multivariate and multinomial regressions were used to analyze factors related to disease prevalence and to health seeking behavior.

**Results:**

One quarter of the children had experienced fever in the past two weeks, 12.0 % had diarrhea and 6.7 % experienced ARI. Children two years of age and older were less likely to experience morbidity than children under one year [OR_fever_ = 0.77, 95 % CI 0.61-0.96; OR_diarrhea_ = 0.26, 95 % CI 0.18-0.37; OR_ARI_ = 0.60 95 % CI 0.41-0.89]. Children aged two and older were more likely than children under one to receive no care or to receive care at home, rather than to receive care at a facility [RRR_diarrhea_ = 3.47, 95 % CI 1.19-10.17 for “No care”]. Children living with an educated caregiver were less likely to receive no care or home care rather than care at a facility as compared to those who lived with an uneducated caregiver [RRR_diarrhea_ = 0.28, 95 % CI 1.10-0.79 for “No care”]. Children living in the wealthiest households were less likely to receive no care or home care for fever as compared to those who lived poorest households. Children living more than 1 km from health facility were more likely to receive no care or to receive home care for diarrhea rather than care at a facility as compared to those living less than 1 km from a facility [RRR_diarrhea_ = 3.50, 95 % CI 1.13-10.82 for “No care”]. Finally, caregivers who lived with more than one child under age five were more likely to provide no care or home care rather than to seek treatment at a facility as compared to those living with only one child under five.

**Conclusions:**

Our results suggest that child age, caregiver education attainment, and household wealth and location may be associated with childhood illness and care seeking behavior patterns. Interventions should be explored that target children and caregivers according to these factors, thereby better addressing barriers and optimizing health outcomes especially for children at risk of dying before the age of five.

## Background

Although global under-five mortality has fallen by almost half (48 %) since 1990, sub-Saharan Africa still experiences unacceptably high child mortality rates, with one in eight children dying before reaching five years of age [1]. A large proportion of these deaths are due to preventable diseases, with diarrhea, pneumonia, and malaria accounting for almost half of all child deaths [2].

Health system strengthening initiatives in Tanzania have contributed to the country’s achievement of the highest density of primary health care facilities in Africa [3]. These efforts have also played a major role in reducing child mortality in Tanzania, placing the country on a trajectory to achieve the Millennium Development Goal 4 of reducing of under-five mortality rate by two-thirds between 1990 and 2015 [4, 5]. However, according to the 2010 Tanzania Demographic and Health Survey (DHS) report, one out of 20 children still dies before his/her first birthday, and one out of 12 dies before his/her fifth birthday [6]. Despite the high density of health care facilities, utilization of life-saving health services remains low, especially in rural areas [7, 8].

Characteristics of caregivers as well as their children have been shown to influence patterns and differences in utilization of health services in Africa and Asia [9–15]. Gender-based discrimination, for example, has been shown to privilege male children in caregiver health-seeking behavior, mainly in South Asia and China but also in Africa [12]. In addition, caregivers with lower socioeconomic status tend to seek and utilize health services for their children less frequently than their wealthier counterparts, likely due to their lower ability to afford services and costs associated with seeking care [9–15]. A multi-country analysis of 11 sub-Saharan Africa DHS data shown that higher level of education of the caregiver was associated with selection of medical centre, pharmacies and home care as compared to no treatment [16]. Another study in Bangladesh found that caregivers with little or no education seek care less frequently than those who are educated [11]. In Tanzania, ill children living in households with multiple children under the age of five are less likely to receive care than those living in households with only one child under the age of five [17]. Finally, fostered children may be less likely to receive health care than their peers, based on evidence for higher mortality among children with a lower degree of biological relatedness to their caregiver in Uganda [18].

Ultimately, children whose caregivers fail to seek health services for them when they become ill are at higher risk for poor health and mortality. As such, it is important to understand determinants and barriers to health service utilization [16, 19]. This study aims to assess the prevalence of fever, diarrhea, and acute respiratory infections (ARI) for children under-five years of age and to identify the determinants of health seeking behavior on their behalf among caregivers in rural Tanzania.

## Methods

### Population characteristics and setting

Data for the present study were collected in the context of the Connect Project. The Connect Project is a randomized cluster trial that investigates the effect of introducing paid community health workers on reducing infant and child mortality and improving maternal health through provision of preventative, promotional, and some curative health services. The intervention aims to address the human resource and service gap between health facilities and households/communities. More detailed information about the project is available in a previous publication [20].

The Connect Project is located in three districts in Tanzania. Kilombero and Ulanga districts are in an isolated area of the Morogoro Region of Tanzania that lies between 200–400 kilometers from Dar es Salaam. Rufiji district is located in Tanzania’s coastal Pwani region. All three districts are predominantly rural and impoverished, with poor transportation and infrastructure. Most inhabitants rely on subsistence agriculture, though there is some fish and wood trade in the Rufiji district.

In these three districts, the Ifakara Health Institute (IHI) maintains two Health and Demographic Surveillance Systems (HDSS) for monitoring mortality and fertility. In 2011, the two HDSS covered a total population of approximately 370,000 people. One fourth were women of reproductive age and 15 % were under five years of age. The under-5 mortality rates were about 61.4 deaths per 1,000 live births in Rufiji in 2012, while the rates were much higher in Ifakara: about 66.6/1000 in Ifakara rural and 78.0/1,000 in Ifakara urban in 2011 [21, 22]. Major causes of mortality for children under five in these regions include malaria, ARI, diarrheal disease, prematurity and low birth weight, and birth injuries and asphyxia [23, 24]. Further details about these HDSS are available in existing publications [25, 26].

### Data sources

This paper uses data from a cross-sectional household survey conducted in May and June of 2011 in the Kilombero, Ulanga, and Rufiji districts of Tanzania. The survey collected baseline data for the Connect project, documenting health seeking behaviors during pregnancy, intrapartum care and immediate newborn care, health service accessibility and utilization of health care. Participants in the study were all women of reproductive age (15–49 years) living in 2,183 selected households in the three study districts. A total of 3,127 women of reproductive age were interviewed [27].

Households for the survey were randomly selected from a list of all HDSS households using probability proportional to size (PPS). In each of the households sampled, all women of reproductive age (15–49 years) were eligible to be interviewed. A woman over 49 years of age was interviewed only if she took care of at least one child less than five years of age in order to obtain information on health and health service utilization pattern for the child [28].

The objective of the survey was to gather baseline maternal and child health indicators for the Connect Project. The present analysis uses survey data on pregnancy history, maternal and child health care and socioeconomic characteristics. Respondents provided information on recent childhood illness with diarrhea, fever, cough, and difficulty of breathing. The prevalence of ARI was estimated by asking the respondent whether the child had a cough accompanied by difficulty breathing.

### Data analysis

Data used in this analysis are from interviews with 1,643 women who were caregivers for 2,077 children under five years old. For each child, proportions were calculated regarding whether he/she was sick with diarrhea (yes or no), fever (yes or no), or ARI (yes or no) within the last two weeks of the interview, whether treatment was sought (yes or no), and where treatment was sought (at home or outside home: health facility or pharmacy or dugs-shop). Only one caregiver sought traditional care for a sick child who had fever and also ARI and this child was removed from the analysis.

The disease prevalence outcome (yes or no) was estimated in terms of odds ratios (OR) that were adjusted for each covariate using multivariate logistic regression analyses. The health-seeking behavior outcome (no care, home care, or facility care) was estimated in terms of relative risk ratios (RRR) that were adjusted for each covariate using multinomial logistic regression analyses. All standard errors were adjusted for clustering at the household level by introducing a household–level random intercept. Computations were conducted using the STATA 12.1 [29].

The analysis examined child, caregiver, household, and contextual factors associated with the odds of experiencing fever, diarrhea and ARI, and of caregiver seeking care during those episodes of illness for children under five years old. Effects of variables significant in the bivariate regressions (p-value < 0.20) and/or shown to be significant in previous studies [12, 13, 18, 30] were included in multivariate models to assess the relative association of individual, household, and village factors with likelihood of experiencing illness and of not receiving care or receiving care at home or in a health facility.

The following predictors of disease prevalence and health care-seeking behavior were selected based on the literature: demographic characteristics of children (sex; age group: less than one year old, between one year and two years old, and more than two years old; and status within the household: fostered or not); characteristics of caregivers (age group: 15–24, 25–34 years and 35 years and older; educational attainment: (no education, primary, and secondary or higher level of education); marital status (married, divorced, widowed or single); religion (Muslim, Christian, or other); ethnic group; relationship with the head of household; district of residency (Kilombero, Ulanga, and Rufiji), and place of residence (rural and semi-urban). Rufiji was used as the reference group for analysis because its geographic characteristics and socio-economic profile are distinct from those of Kilombero and Ulanga.

Relative economic status of households was quantified through principle component analysis (PCA) of indicators such as water and sanitation facilities and household size [31]. PCA scores were ordered and used to divide the households into five index groups. Children in the fifth quintile (wealthiest) were compared with those in first quintile (poorest) to assess whether wealth is a predictor of seeking care during episodes of illness. Using the household-level data, we calculated the distance from each household to closest health facility (less than 1 km, between 1 and 2 km, between 2 and 4 km and 4 km and more) to analyze the extent to which distance to closest health facility impacts seeking care during episodes of illness.

### Ethical consideration

The study was approved by the Columbia University Medical Center Institutional Review Board (Protocol AAAF3452), by the National Institute for Medical Research of the Medical Research Coordinating committee (NIMR-CC) (NIMR/HQ/R.8a/Vol.IX/1203) and by the Ifakara Health Institute’s Institutional Review Board (IHI/IRB/No. 16–2010). Participation in the survey was entirely voluntary and participants signed an informed consent prior to the onset of interviewing. For each participant less than 18 years of age, consent was sought from their respective parent/guardian.

## Results

### Participant characteristics

The analysis included 1,643 women who reported living with 2,077 children under five years old. More than half (51.6 %) were male and 48.4 % were female. The mean age of the children was 2.1 years (SD = 1.3; range: 0–59 months) and only 5.0 % of children were fostered.

The mean age of the caregivers was 30.0 years (SD = 8.4; range: 15–70). Most women (81.4 %) were married or living with a partner as if married. Seventy-one percent had completed the primary level of education while 4.7 % attended the secondary level of education and 24.3 % had no formal schooling. More than three-quarters of caregivers (76.0 %) lived with only one child under five years old while one-quarter (24.0 %) lived with two or more children under five years old. Caregivers living in the poorest households (1st wealth quintiles) comprised 21.6 % of the repondents and 13.5 % lived in the wealthiest households (5th wealth quintiles). About 7.3 % of caregivers did not provide information regarding economic status of their households. Nearly one quarter of the caregivers (24.5 %) lived in Rufiji while 59.2 % lived in Kilombero and only 16.3 % lived in Ulanga district. About 29.2 % of children lived within 1 km from a health facility while 24.6 % lived more than 4 km from a health facility (Table 1).

**Table 1:** Population characteristics in Rufiji, Ulanga and Kilombero districts of Tanzania, 2011 (children, n = 2,077; caregivers, n = 1,643)

Characteristic	n (% or SD)
Child characteristics	
Gender	
Male	1084 (52.19)
Female	993 (47.81)
Mean age (years)	2.09 (1.32)
0 - 1 year	876 (42.18)
1 - 2 years	382 (18.39)
2 - 5 years	819 (39.43)
Foster status	
Not foster child	1973 (94.99)
Foster child	104 (5.01)
Caregiver characteristics	
Mean age (years)	29.97 (8.39)
15 - 24 years	516 (31.41)
25 - 34 years	622 (37.86)
≥35 years	505 (30.74)
Educational achievement	
None	399 (24.28)
Primary	1167 (71.03)
Secondary and plus(a)	77 (4.69)
Marital status	
Married(b)	1338 (81.44)
Unmarried	305 (18.56)
Religion	
Muslim	858 (52.22)
Christian	706 (42.97)
Traditional	79 (4.81)
Ethnic Group	
Ndengereko	272 (16.56)
Pogoro	237 (14.42)
Sukuma	150 (9.13)
Ngindo	233 (14.18)
Other	751 (45.71)
Relationship to head of household	
Wife	948 (57.70)
Head of household	77 (4.6)
Daughter	299 (18.20)
Daughter-in-law	123 (7.49)
Other	196 (11.93)
Number of children under age 5 in household	
One child under 5	1248 (76.56)
Two children under 5	357 (21.73)
Three children under 5	38 (2.31)
Wealth quintiles	
First Quintile (Poorest)	355 (21.61)
Second Quintiles	422 (25.68)
Third Quintiles (Middle)	287 (17.47)
Fourth Quintiles	237 (14.42)
Fifth Quintiles (Wealthiest)	222 (13.51)
NR	120 (7.30)
District	
Rufiji	403 (24.53)
Kilombero	973 (59.22)
Ulanga	267 (16.25)
Residence	
Rural	1340 (81.56)
Urban/semi-urban	303 (18.44)
Nearest health facility characteristics	
Ownership	
Government	767 (46.68)
Private	876 (53.32)
Type	
Dispensary	1222 (74.38)
Health center	203 (12.36)
Hospital	218 (13.27)
Distance to village	
Mean (SD) = 3.001 (3.328)	
<1 km	479 (29.15)
1-2.99 km	613 (37.31)
> = 3 km	551 (33.54)

ARI = Acute respiratory infection (cough accompanied with difficulty of breathing

NR = Non-response

(a) Defined as ordinary or advanced secondary school

(b) Defined as married or living with a partner as if married

### Illness prevalence and health seeking care

Approximately one quarter of children (24.8 %, n = 515) had fever within the last two weeks of the interview, 12.0 % (n = 250) had diarrhea and 6.7 % (n = 139) had ARI.

Regarding parental care seeking, most children with fever (91.5 %) and ARI (92.1 %) received care while this proportion decreased to 73.2 % of children with diarrhea. About three-quarter of children with fever (74.3 %) and ARI (77.3 %) were initially treated at a health facility (dispensary, health center or hospital) while this proportion was only 23.0 % for children with diarrhea. About 60.1 % of children with diarrhea were treated at first at home while this proportion was only 5.7 % for children with fever and 3.1 % for children with ARI. Only 1 child with concomitant fever and ARI were treated outside the home with traditional care. Nearly 45 % of all children with fever or ARI and 41 % of children with diarrhea were treated in a public facility.

The mean duration of diarrhea was 4.5 days (SD = 4.1; range: 1–30). Of the children treated at home, 78.7 % received oral rehydration therapy (ORS) while 20.8 % received a home rehydration solution (HRS). During illness episodes, 44.4 % and 32.2 % of children ate and drank about the same as usual, 42.0 % and 33.2 % of children ate and drank less than usual, 4.4 % and 26.0 % of children ate and drank more than usual and 3.2 % and 1.2 % of children ate and drank nothing (Table 2).

**Table 2:** Illness prevalence and health seeking behaviors (Children, n = 2077)

Characteristic			n (% or SD)
*A. Illness prevalence*			
Fever			515 (24.80)
Diarrhea			250 (12.04)
ARI			139 (6.69)
*B. Seeking care*			
Fever			471 (91.46)
Diarrhea			183 (73.20)
ARI			128 (92.09)
*C. Place of care (first option)*			
	*Fever (n = 471)*	*Diarrhea (n = 183)*	*ARI (n = 128)*
Home	27 (5.73)	110 (60.11)	4 (3.12)
Dispensary	234 (49.68)	32 (17.49)	73 (57.03)
Health Center	78 (16.56)	17 (9.29)	17 (13.28)
Hospital	38 (8.07)	4 (2.19)	9 (7.03)
Pharmacy/drug shop	87 (18.47)	20 (10.93)	22 (17.19)
Traditional	1 (0.21)	0 (0.00)	1 (0.78)
Other (CHW, Neighbor)	6 (1.27)	0 (0.00)	2 (15.63)
*D. Ownership of Health facility*			
	*Fever (n = 444)*	*Diarrhea (n = 73)*	*ARI (n = 124)*
Public	195 (43.92)	30 (41.10)	55 (44.35)
Private	198 (44.59)	43 (58.90)	53 (42.74)
Don’t Know	51 (11.49)	0 (0.00)	16 (12.9)
*E. Diarrhea treatment at home (n = 183)*			
Oral Rehydration Salts (ORS)			144 (78.69)
Home Rehydration Solution (HRS)			38 (20.77)
Only Home Rehydration Solution (HRS)			12 out of 39 (30.77)
*F. Feeding during diarrhea (n = 250)*			
*Eating*			
Much less			32 (12.80)
Somewhat less than usual to eat			73 (29.20)
About the same as usual to eat			111 (44.40)
More than usual to eat			11 (4.40)
Nothing to eat			8 (3.20)
Non Response			15 (6.00)
*Drinking*			
Much less			30 (12.00)
Somewhat less than usual to drink			53 (21.20)
About the same as usual to drink			83 (32.20)
More than usual to drink			65 (26.00)
Nothing to drink			3 (1.20)
Non Response			16 (6.40)
*G. Lasting (days)*			
Mean age (days)			4.45 (4.12)
1			23 (9.20)
2_3			123 (49.20)
> = 4			104 (41.60)

### Determinants of illness prevalence

Children age one year and older were less likely to experience diseases than children under one year old in unadjusted bivariate analysis as well as multivariate analysis adjusted for other covariates. Oldest children aged 2 to 5 years had lower odds of experiencing a fever, diarrhea, or ARI episode than children under 1 [OR_fever_ = 0.77, 95 % CI 0.61-0.96; OR_diarrhea_ = 0.26, 95 % CI 0.18-0.37; OR_ARI_ = 0.60, 95 % CI 0.41-0.89]. Children of caregivers aged 25 to 34 were less likely to have diarrhea reported as an illness episode as compared to children of younger caregivers (below 25 years) [OR_diarrhea_ = 0.68, 95 % CI 0.48-0.96]. Children of educated caregivers were more likely to have diarrhea reported as an illness episode as compared to children of not educated caregivers. Children who lived in the wealthiest household were less likely experience diseases compared to children who lived in the poorest household [OR_fever_ = 0.65, 95 % CI 0.43-0.99]. Children of caregivers who lived with more than one child under five were less likely to experience diseases compared to children of caregivers who lived with only one child under five [OR_fever_ = 0.64, 95 % CI 0.50-0.82; OR_ARI_ = 0.61, 95 % CI 0.40-0.95]. Children who lived more than 1 km from a health facility were less likely to experience diarrhea compared to children who lived within 1 km in unadjusted as well as adjusted analysis for other covariates.

Illness experiences were not significantly associated with child’s gender or status within the household (fostered or not), or with caregiver marital status, religion, ethnic group, relationship to the head of household, place of residence (rural or semi-urban) or district of residence. Models with interaction terms did not show a significant association between maternal education attainment and maternal age, household wealth or distance to nearest health facility (Table 3).

**Table 3:** Unadjusted and adjusted odds ratios (OR) and 95 % confidence intervals (CI) estimated through bivariate and multivariate logistic regression of illness prevalence in Rufiji, Ulanga and Kilombero districts of Tanzania, 2011

Illness prevalence	Unadjusted OR (95%CI)			Adjusted OR (95%CI)		
	Fever	Diarrhea	ARI	Fever	Diarrhea	ARI
Child characteristics						
Male	Ref.	Ref.	Ref.	—	—	—
Female	0.91 (0.74-1.11)	1.00 (0.76-1.30)	1.09 (0.76-1.55)	—	—	—
Child age						
0 - 1 year	Ref.	Ref.	Ref.	Ref.	Ref.	Ref.
1 - 2 years	1.01 (0.77-1.32)	0.47 (0.32-0.68)***	0.73 (0.45-1.17)	1.01 (0.76-1.33)	0.50 (0.34-0.74)**	0.72 (0.44-1.16)
2 - 5 years	0.75 (0.61-0.93)*	0.24 (0.17-0.34)***	0.57 (0.39-0.84)**	0.77 (0.61-0.96)*	0.26 (0.18-0.37)**	0.60 (0.41-0.89)*
Foster child status						
Non-foster child	Ref.	Ref.	Ref.	—	—	—
Foster child	1.24 (0.80-1.94)	0.95 (0.51-1.79)	1.52 (0.74-3.14)	—	—	—
Caregiver characteristics						
Caregiver age						
15 - 24 years	Ref.	Ref.	Ref.	Ref.	Ref.	
25 - 34 years	0.82 (0.63-1.05)	0.52 (0.38-0.72)***	0.83 (0.54-1.28)	0.88 (0.66-1.16)	0.68 (0.48-0.96)*	—
≥35 years	1.01 (0.78-1.32)	0.49 (0.34-0.69)***	0.88 (0.56-1.40)	1.06 (0.78-1.46)	0.74 (0.48-1.14)	—
Educational achievement						
No education	Ref.	Ref.	Ref.		Ref.	
Primary	1.04 (0.82-1.33)	1.49 (1.06-2.09)*	1.19 (0.77-1.83)	—	*1.53 (0.99-2.36)^*	—
Secondary and plus (a)	1.21 (0.71-2.10)	2.31 (0.123-4.35)**	1.19 (0.48-2.94)	—	*1.97 (0.94-4.12)^*	—
Marital status						
Married (b)	Ref.	Ref.	Ref.	—	Ref.	Ref.
Unmarried	1.18 (0.90-1.55)	1.29 (0.92-1.82)	1.41 (0.90-2.22)	—	0.93 (0.61-1.40)	1.11 (0.64-1.91)
Religion						
Muslim	Ref.	Ref.	Ref.	Ref.	Ref.	Ref.
Christian	0.80 (0.64-0.99)*	0.95 (0.71-1.28)	0.77 (0.52-1.12)	0.88 (0.67-1.17)	0.74 (0.52-1.07)	0.85 (0.52-1.40)
Traditional/Other	0.66 (0.40-1.10)	1.51 (0.86-2.63)	0.84 (0.37-1.92)	0.72 (0.34-1.51)	1.83 (0.71-4.71)	0.77 (0.26-2.26)
Ethnic Group						
Ndengereko	Ref.	Ref.	Ref.	Ref.	Ref.	Ref.
Pogoro	0.74 (0.51-1.07)	1.40 (0.82-2.40)	0.81 (0.43-1.52)	1.07 (0.63-1.81)	1.49 (0.67-3.31)	1.42 (0.61-3.32)
Sukuma	*0.68 (0.45-1.03)^*	*1.64 (0.94-2.85)^*	0.79 (0.40-1.54)	1.26 (0.62-2.55)	1.29 (0.48-3.44)	1.52 (0.53-4.37)
Ngindo	0.76 (0.52-1.10)	*1.55 (0.92-2.63)^*	0.57 (0.29-1.12)	0.95 (0.57-1.58)	1.40 (0.66-3.00)	0.87 (0.37-2.00)
Other	0.81 (0.61-1.07)	*1.48 (0.96-2.28)^*	0.72 (0.44-1.18)	1.19 (0.77-1.82)	1.55 (0.79-3.06)	1.26 (0.62-2.56)
Relationship to head of household						
Head of household/Wife	Ref.	Ref.	Ref.	Ref.	Ref.	Ref.
Daughter/Daughter in law	1.19 (0.94-1.51)	1.88 (1.37-2.57)***	1.38 (0.91-2.09)	1.13 (0.85-1.50)	1.23 (0.80-1.88)	1.16 (0.71-1.89)
Other	0.80 (0.55-1.15)	1.70 (1.10-2.64)***	1.58 (0.90-2.77)	0.77 (0.52-1.14)	1.31 (0.78-2.19)	1.45 (0.81-2.61)
Number of children under age 5 in household						
One child under 5	Ref.	Ref.	Ref.	Ref.	Ref.	Ref.
More than one child under 5	0.64 (0.51-0.81)***	0.71 (0.52-0.96)*	0.63 (0.41-0.97)*	0.64 (0.50-0.82)***	0.78 (0.56-1.08)	0.61 (0.40-0.95)*
Wealth Index						
Poorest	Ref.	Ref.	Ref.	Ref.	Ref.	Ref.
2nd Quintiles	0.62 (0.46-0.83)**	0.59 (0.39-0.90)*	0.56 (0.33-0.96)*	0.66 (0.48-0.89)**	0.58 (0.37-0.92)*	0.65 (0.37-1.13)
3rd Quintiles	0.65 (0.46-0.91)*	0.85 (0.55-1.32)	0.43** (0.23-0.80)	0.68 (0.46-0.99)*	0.76 (0.47-1.23)	0.48 (0.24-0.98)*
4th Quintiles	*0.74 (0.52-1.05)^*	1.15 (0.72-1.83)	0.84 (0.48-1.47)	0.75 (0.52-1.10)	0.90 (0.53-1.53)	0.97 (0.53-1.76)
Wealthiest	0.66 (0.45-0.95)*	0.95 (0.59-1.54)	0.77 (0.43-1.40)	0.65 (0.43-0.99)*	0.73 (0.41-1.33)	0.86 (0.45-1.66)
NR	0.56 (0.36-0.88)*	0.81 (0.47-1.41)	0.80 (0.40-1.62)	*0.66 (0.41-1.07)^*	0.66 (0.35-1.27)	0.94 (0.42-2.12)
District						
Rufiji	Ref.	Ref.	Ref.	Ref.	Ref.	Ref.
Kilombero	0.69** (0.54-0.88)	*1.34 (0.95-1.89)^*	0.59 (0.39-0.89)*	0.80 (0.53-1.20)	1.91 (0.64-2.21)	0.58 (0.31-1.12)
Ulanga	0.68 (0.49-0.93)*	1.25 (0.79-1.97)	0.68 (0.39-1.20)	0.84 (0.52-1.41)	1.02 (0.51-2.06)	0.74 (0.33-1.68)
Place of residence						
Rural	Ref.	Ref.	Ref.	—	—	—
Urban/semi-urban	0.93 (0.70-1.23)	0.84 (0.57-1.25)	1.08 (0.68-1.71)	—	—	—
Nearest health facility characteristics						
Ownership						
Government	Ref.	Ref.	Ref.	Ref.	—	
Private	1.27 (1.02-1.57)*	1.12 (0.85-1.49)	1.23 (0.85-1.7)	1.28 (1.03-1.59)*	—	—
Type						
Dispensary	Ref.	Ref.	Ref.	—	Ref.	—
Health center/Hospital	0.90 (0.70-1.15)	1.26 (0.92-1.72)	0.96 (0.63-1.45)	—	1.18 (0.80-1.72)	—
Distance to household						
<1 km	Ref.	Ref.	Ref.	—	Ref.	
1-1.99 km	1.01 (0.75-1.37)	0.84 (0.57-1.23)	0.95 (0.57-1.60)	—	0.75 (0.49-1.13)	—
2-3.99 km	1.06 (0.80-1.43)	0.51 (0.34-0.78)**	0.99 (0.60-1.66)	—	0.51 (0.32-0.80)**	—
> = 4 km	1.04 (0.78-1.39)	1.06 (0.73-1.52)	1.08 (0.66-1.78)	—	1.16 (0.75-1.81)	—

Variables significant at 20 % in bivariate analysis were included in multivariate analysis

ARI = Acute respiratory infection (cough accompanied with difficulty of breathing)

(a) Defined as ordinary or advanced secondary school

(b) Defined as married or living with a partner as if married

(c) Models with interaction terms did not show a significant association between maternal education attainment and maternal age, household wealth or distance to nearest health facility.

***p < 0.001; **p < 0.01; *p < 0.5

### Determinants of health seeking care

Due to the small number of children who experienced ARI (n = 139), we limited multivariate multinomial logistic regression analysis to episodes of fever and diarrhea. Adjusting for other covariates, oldest children (aged 2 to 5 years) with diarrhea were more likely to receive home care or no care rather than facility care as compared to young children (<1 year old) [RRR_diarrhea_ = 3.47, 95 % CI 1.19-10.17 for “No care”].

Caregivers of age 35 and above, as compared to younger caregivers (below 25 years), were more likely to provide home care or no care for fever rather than to seek treatment at a facility [RRR_fever_ = 2.49, 95 % CI 1.00-6.25 for “No care”]. Educated caregivers were less likely to provide no care for diarrhea rather than seeking treatment at the facility as compared to uneducated caregivers [RRR_diarrhea_ = 0.28, 95 % CI 0.10-0.79 for “Primary level of education”].

Christian children, as compared to Muslim children, were more likely to receive home care or no care for diarrhea rather than to receive care at the facility [RRR_diarrhea_ = 2.70, 95 % CI 1.11-6.59 for “No care”; RRR_diarrhea_ = 2.50, 95 % CI 1.11-5.65 for “Home care”]. Sukuma children were generally more likely to receive home care or no care rather than facility care as compared to children of other ethnicities.

Caregivers who lived with two or more children under five, as compared to caregivers who lived with only one child, were more likely to provide home care or no care for fever rather than seeking treatment at the facility [RRR_fever_ = 2.27, 95 % CI 1.07-4.72 for “No care”]. Daughters or daughters-in-law and others caregivers, as compared to women head of household or wives of the heads of household, were generally more likely to provide home care or no care rather than seeking care at the facility.

Children who lived in wealthiest households, as compared to those who lived in the poorest household, were less likely to receive home care or no care for fever rather than facility care [RRR_fever_ = 0.25, 95 % CI 0.06-1.00 for “3rd quintile; RRR_fever_ = 0.22, 95 % CI 0.04-1.17, p = 0.076 for “5th quintile”]. Children who lived in Kilombero and Ulanga, as compared to those who lived in Rufiji district, were more likely to receive home care or no care for fever rather than facility care [RRR_fever_ = 6.95, 95 % CI 1.00-48.41 for “Kilombero”]. Children who lived more than 1 km from a health facility, as compared to those who lived within 1 km from a health facility, were more likely to receive home care or no care for diarrhea rather than facility care [for “1-2 km”: RRR_diarrhea_ = 3.50, 95 % CI 1.13-10.81 for “No care”; RRR_diarrhea_ = 2.89, 95 % CI 1.04-8.02 for “Home care”].

Health-care seeking was not significantly associated with child’s gender or status within the household (fostered or not), caregiver’s marital status, or with characteristics’ of nearest facility (ownership or type). Models with interaction terms did not show a significant association between maternal educational attainment and maternal age, household wealth, or distance to nearest health facility (Table 4).

**Table 4:** Adjusted relative risk ratios (RRR) and 95 % confidence intervals estimated through multivariate multinomial logistic regression of health-seeking behavior by child and caregiver characteristics in Rufiji, Ulanga and Kilombero districts of Tanzania, 2011

Care seeking for illness	Fever (n = 514)		Diarrhea (n = 250)	
Level of treatment received	No care received vs. Facility care	Home care vs. Facility care	No care received vs Facility care	Home care vs. Facility care
Child characteristics				
Child age				
0 ≤ 1 year	Ref.	Ref.	Ref.	Ref.
1 - 2 years	0.63 (0.23-1.70)	0.78 (0.21-2.85)	0.81 (0.27-2.47)	1.14 (0.45-2.86)
2 - 5 years	0.74 (0.35-1.57)	1.14 (0.50-2.61)	3.47 (1.19-10.17)*	2.02 (0.75-5.42)
Caregiver characteristics				
Caregiver age				
15 - 24 years	Ref.	Ref.	Ref.	Ref.
25 - 34 years	0.56 (0.19-1.62)	0.80 (0.24-2.65)	0.52 (0.19-1.41)	0.52 (0.19-1.47)
≥35 years	2.49 (1.00-6.25)*	1.40 (0.49-4.01)	0.40 (0.14-1.19)	0.67 (0.26-1.71)
Educational achievement				
No education	—	—	Ref.	Ref.
Primary	—	—	0.28 (0.10-0.79)*	0.54 (0.21-1.37)
Secondary and plus (a)	—	—	0.29 (0.05-1.92)	1.09 (0.23-5.21)
Religion				
Muslim	Ref.	Ref.	Ref.	Ref.
Christian	0.62 (0.27-1.42)	0.73 (0.21-2.61)	2.70 (1.11-6.59)*	2.50 (1.11-5.65)*
Traditional/Other	1.24 (0.24-6.37)	0.97 (0.12-7.89)	0.07 (0.01-0.63)*	0.63 (0.10-3.99)
Ethnic Group				
Ndengereko	Ref.	Ref.	Ref.	Ref.
Pogoro	1.37 (0.15-12.33)	3.54 (0.51-24.75)	0.77 (0.17-3.42)	1.05 (0.23-4.66)
Sukuma	2.03 (0.19-22.69)	*6.79 (0.83-55.63)^*	1.19 (0.21-6.78)	1.44 (0.22-9.56)
Ngindo	1.09 (0.15-8.13)	1.66 (0.29-9.48)	0.54 (0.11-2.52)	0.49 (0.11-2.24)
Other	1.23 (0.16-9.11)	1.08 (0.21-5.65)	0.25 (0.06-1.00)*	*0.33^ (0.09-1.22)*
Relationship to head of household				
Head of household/Wife	Ref.	Ref.	Ref.	Ref.
Daughter/Daughter in law	*2.13 (0.89-5.07)^*	1.13 (0.44-2.89)	1.40 (0.58-3.45)	1.00 (0.44-2.29)
Other	3.07 (1.01-9.36)*	0.40 (0.09-1.90)	1.11 (0.30-4.14)	1.82 (0.59-5.60)
Number of children under age 5 in household				
One child under 5	Ref.	Ref.	Ref.	Ref.
More than one child under 5	2.27 (1.09-4.75)*	2.14 (0.76-6.03)	0.85 (0.31-2.34)	1.72 (0.76-3.87)
Wealth Index				
Poorest	Ref.	Ref.		
2nd Quintiles	0.62 (0.22-1.69)	0.71 (0.21-2.41)	—	—
3rd Quintiles	0.25 (0.06-1.00)*	0.53 (0.07-3.88)	—	—
4th Quintiles	0.43 (0.10-1.73)	0.88 (0.14-5.45)	—	—
5th Quintiles	*0.22 (0.04-1.17)^*	0.62 (0.08-4.91)	—	—
NR	0.97 (0.33-2.87)	0.27 (0.03-2.92)	—	—
District				
Rufiji	Ref.	Ref.		
Kilombero	6.95 (1.00-48.41)*	1.15 (0.15-8.83)	—	—
Ulanga	2.19 (0.26-18.70)	0.43 (0.06-2.91)	—	—
Place of residence				
Rural	Ref.	Ref.	Ref.	Ref.
Urban/semi-urban	0.75 (0.23-2.50)	2.34 (0.73-7.54)	3.01 (1.00-9.20)*	*2.52 (0.85-7.45)^*
Nearest health facility characteristics				
Distance to household				
<1 km	Ref.	Ref.	Ref.	Ref.
1-1.99 km	0.54 (0.19-1.50)	0.64 (0.15-2.73)	3.50 (1.13-10.81)*	2.89 (1.04-8.02)*
2-3.99 km	0.92 (0.35-2.42)	0.81 (0.21-3.19)	2.20 (0.66-7.32)	1.39 (0.48-4.04)
> = 4 km	0.47 (0.16-1.34)	2.10 (0.55-8.01)	1.05 (0.38-2.91)	0.63 (0.26-1.55)

***p < 0.001; **p < 0.01; *p < 0.5; ^P < 0.10

Variables significant at 20 % in bivariate analysis were included in multivariate analysis

(a) Defined as ordinary or advanced secondary school

(b) Defined as married or living with a partner as if married

(c) Models with interaction terms did not show a significant association between maternal education attainment and maternal age, household wealth or distance to nearest health facility.

## Discussion

This cross-sectional analysis of child and caregiver characteristics aimed to explore illness prevalence for children under five years old as well as also patterns in health-seeking behavior. This was undertaken with the hope of understanding barriers to treatment for diarrhea, one of the leading causes of under-five mortality in Tanzania, as well as symptoms (i.e., fever and cough accompanied with difficulty breathing) that may suggest more serious illness such as malaria or pneumonia.

The prevalence of childhood illnesses (fever, diarrhea and ARI) in our study was comparable to national estimates reported in the recent DHS report [32]. Our findings of disease prevalence by age group of child were also comparable to a recent analysis of national DHS data [17], which showed that fever and diarrhea were more common among youngest children (1 year old and below) as compared to oldest children (2 years old and above). Educated women reported a higher frequency of episodes of fever, diarrhea and ARI than uneducated women; a finding that is consistent with national DHS data [17].

Fever and ARI were more frequently treated at a facility, while diarrhea was usually treated at first at home. The use of the simple and standard treatment for diarrhea treatment (ORS or HRS) remains sub-optimal in many countries including Tanzania, where diarrhea accounts for many child deaths globally [33]. In our study, almost all children (99 %) treated at home received ORS or HRS.

The type of health care sought was significantly associated with child age and with caregiver education attainment, religion, ethnic group, number of children under five years old in the household, wealth quintile, district of residence and distance from household to nearest health facility. Caregivers were more likely to seek facility care versus no care for children under one year with diarrhea, and less likely to seek facility care versus no care for children one year and older with fever. This was consistent with similar studies in Tanzania and Kenya which assessed health seeking behavior for children under five with fever and/or cough [13, 18]. However, child gender was not found to be associated with differences in health seeking behavior. While gender discrimination is seen within health seeking behavior in other Sub-Saharan African countries [12], our results imply that health-seeking behavior is not influenced by gender of the child in rural Tanzania. This is confirmed by other studies in Tanzania [17], Kenya [13, 14] and Sierra Leone [34].

Regarding characteristics of caregivers, educational achievement was a strong determinant of health care seeking such that children of educated caregivers would receive more facility care than those of uneducated caregivers. This result was comparable to the findings from early studies in Africa and Asia [14][11, 17, 35] although a recent study from Sierra Leone found no relationship between education and health seeking behavior [34]. Caregiver’s age was also found to influence health seeking behavior for fever by influencing caregiver perception of illness. Children of older caregivers were significantly less likely to have a diarrhea illness reported than their peers. It is possible that diarrhea events among children were not perceived as illness episodes by older or uneducated caregivers. Caregivers with no formal education had significantly lower rates of sensitivity in diagnosing child diarrhea in a study of maternal recognition of childhood diarrhea in Nigeria [36].

Additionally, relationship to head of household was associated with health seeking behavior. Caregiver’s relationship to the head of the household is an important predictor of health seeking behavior; caregivers with more decision-making power (the head of the household or the wife of the head of household) are more like to seek facility care than those with a lower status in the household [37].

Wealth quintile of the household was seen to significantly influence health-seeking behavior. During the episode of fever, caregivers in the wealthiest quintiles were more likely to seek care at the health facility rather than not to provide care as those in the poorest quintiles. These results imply that wealthiest women were more readily seek care at the facility for child illness than poorest women, indicating that financial readiness influences care seeking as shown by previous studies [11, 13–15].

Our study also indicated that children residing in households with two or more children under five were less likely to receive facility care for fever than children residing in households as the only child under age five. Another study in Tanzania had a similar finding [18]. This fact is likely explained by the resource and time constraints associated with caring for multiple young children that serve as a barrier to seeking care.

Health seeking behavior for fever was associated with district of residence; residents of Kilombero and Ulanga were more likely to not seek care or to provide care at home rather than to seek care at the facility, as compared with caregivers in Rufiji. Trends observed in health seeking behavior by ethnic group may explain these district-level patterns. A higher proportion of participants who are residents of Rufiji are Ndengereko ethnicity as compared to Kilombero and Ulanga residents who are predominantly of other ethnicities. Beliefs and practices surrounding health that are associated with ethnic groups or religion also might explain differences in health seeking behavior by district.

Health seeking behavior for diarrhea was inversely associated with distance to health facility. Children who lived near to health facility received more care at the facility than those who lived far from a health facility. These findings are also broadly consistent with other data from sub-Saharan Africa. Several studies have documented an association between distance from health facility to many outcomes, including services utilization and child mortality [38–41].

### Limitations

Our study is subject to limitations posed by self-reported data and a lack of road networks and footpaths data or time-travel data. Therefore, measurement of illness episodes was based on the caregiver’s perception of illness and was not validated clinically. In addition, the accuracy and completeness of reporting on both illness and health-seeking behavior was subject to recall and social desirability bias. Furthermore, important determinants of health seeking behavior such as “real” distance to facility or time to reach the facility were not available. As such, it was not possible to assess with accuracy how the distance and the time-travel from household to closest health facility affected the facility seeking care during childhood illnesses. Further research will utilize GIS-linked individual-level household survey data and road networks and footpaths, which allows to calculate real distances and also time-travel from each household to the closest facility.

Due to limitation of the sample size, factors associated with health seeking behavior for ARI could not be assessed because of the low number of reported episodes of ARI.

## Conclusion

Our study identified factors that contribute to a caregiver’s failure to seek potentially life-saving health care for an ill child under the age of five. Child age was shown to be a determinant of treatment, with older children with diarrhea being less likely to receive facility care. In addition, children taken care of by uneducated women or women caring for more than one child under age five, and those who lived in poorest household or household located far from a health facility were less likely to receive facility care. Based on these results, we suggest that interventions should be implemented that address these patterns in order to optimize health outcomes for children at risk of dying before the age of five.

This study presents an exploratory analysis of the determinants of health seeking behavior. To design appropriate interventions to increase child survival further analysis is needed to assess the relationship between caregiver characteristics, child characteristics, health seeking behavior patterns, and child mortality.
